# Editorial and Miscellaneous

**Published:** 1862-05

**Authors:** 


### EDITORIAL AND MISCELLANEOUS.

*The Sanitary Commission.*—Etertaining at the outset of the present war a "generous confidence" in the government of the country, and knowing something of the gigantic means at its diposal, and the large number of well paid officials whose business it was to render those means efficient, we were among those who doubted the great necessity of the so-called Sanitary Commission. We regretted that its head and front was not, as common sense would have suggested, a practical medical man; and a variety of circumstances, not necessary now to be repeated, conspired to produce the conviction that the organization would ultimate in a failure.

But the immense resources of government were largely depleted by private peculation, as well as by the unexpected magnitude of the war.

Officials were found sometimes overworked, oftener slothful and negligent, and perhaps oftener still, either incompetent or altogether knavish.

The filling up of requisitions from regimental or brigade surgeons by the officials having the matter in charge was, and is, an almost unheard of thing. Whole regiments and even brigades have been kept destitute of the most indispensable medicines. Government hospital transports utterly unprovided with even the cheapest and most abundant of supplies for the sick and wounded.

And this, be it observed, without a shadow of negligencce or want of forethought on the part of the medical men detailed to the charge of them. The poor excuse of difficulty of obtaining these supplies cannot be made by the *head* officials, for the markets are positively glutted with them, and the holders are abundantly anxious to secure even the certificates of indebtedness of "our common Uncle."

The slightest skirmish has about invariably found the official hospital supplies and appliances defficient to a deplorable extent, whilst Fort Donelson and Shiloh, have shown an infini tesimal attenuation of positive necessaries, which would shame the pitiful attempts of the Homœpathists from Hanhemann downward to imitate.

We repeat there is not a shadow of an excuse for the failure of the officials to answer immediately and fully the requisitions made upon them for supplies by the brigade, regimental and hospital surgeons.

But there seems no help for it according to the present order of things, unless, perchance, these central officials be transplanted to some better paying or more honorable positions, and new and active, or at least honest men put in their places. (It is suggested by a friend at our elbow that a dozen foreign missions be created for them, if none are now vacant—and this perhaps would be the cheapest way in the long run.)

Public opinion would not permit the soldier to suffer, although he squandered his hardly earned wages for the sutler's trash, and although the powers that be were incompetent, neglectful or worse.

The Sanitary Commission, a self-constituted committee, was taken up by the profession and the public, and it is but simple justice to say that it has discharged the business assumed and devolved upon it in a way and to an extent which challenges the admiration of the country, as it has secured the unbounded gratitude of the suffering soldiery.

The amount of supplies, positive necessaries, conveniences, little luxuries and comforts, and attention which they have been instrumental in conveying gratuitously to the grand army is truly wonderful, and puts to shame the sleepy or scoundrelly army officials.

As soon as possible it is intended to give some of the details of this immense work and outlay to our readers.

We are proud to chronicle the large number of professional gentlemen who have contributed their personal services gratuitously, and often at considerable cost, aside from time and interference with their current business, to the wounded and sick on the field and in the hospitals. In this respect, also, the Sanitary Commission are entitled to thanks. They have done much to secure the services of competent men, and at the same time to prevent the disabled soldiers from falling into the hands of charlatans and unfeeling experimenters. Our brigade and regimental Surgeons have been overworked and many of them have fallen victims to exposure and fatigue. They have needed, and will continue to require more help from Surgeons in civil practice. We trust that none who can go will be dissuaded by having once satisfied his curiosity, or experienced the hardships and privations of a military visitation. Meanwhile, honor to the Sanitary Commission.

